# Parameter Estimation Error Dependency on the Acquisition Protocol in Diffusion Kurtosis Imaging

**DOI:** 10.1007/s00723-016-0829-x

**Published:** 2016-09-17

**Authors:** Nima Gilani, Paul N. Malcolm, Glyn Johnson

**Affiliations:** 1 Norwich Medical School, University of East Anglia, Bob Champion Research and Educational Building, Room 2.18, James Watson Road, Norwich Research Park, Norwich, NR4 7UQ UK; 2 Norfolk and Norwich University Hospitals, Norwich, UK

## Abstract

Mono-exponential kurtosis model is routinely fitted on diffusion weighted, magnetic resonance imaging data to describe non-Gaussian diffusion. Here, the purpose was to optimize acquisitions for this model to minimize the errors in estimating diffusion coefficient and kurtosis. Similar to a previous study, covariance matrix calculations were used, and coefficients of variation in estimating each parameter of this model were calculated. The acquisition parameter, *b* values, varied in discrete grids to find the optimum ones that minimize the coefficient of variation in estimating the two non-Gaussian parameters. Also, the effect of variation of the target values on the optimized values was investigated. Additionally, the results were benchmarked with Monte Carlo noise simulations. Simple correlations were found between the optimized *b* values and target values of diffusion and kurtosis. For small target values of the two parameters, there is higher chance of having significant errors; this is caused by maximum *b* value limits imposed by the scanner than the mathematical bounds. The results here, cover a wide range of parameters *D* and *K* so that they could be used in many directionally averaged diffusion weighted cases such as head and neck, prostate, etc.

## Introduction

Quantitative diffusion MRI has proved useful in characterizing tumours in a number of different cancers [1, 2], by estimating apparent diffusion coefficient of water molecules assuming the diffusion is Gaussian inside the organ. However, the relationship between the signal (*S*) and acquisition *b* values is not generally Gaussian because of compartmentalization, hindrance, and restriction effects on diffusion [3, 4], or generally complexity of diffusion. As a result, an additional term kurtosis (*K*) is added to the Gaussian model to describe this non-Gaussian behaviour [3, 4]:

1 $$S = S_{0} {\text{e}}^{{ - bD + \frac{{Kb^{2} D^{2} }}{6}}} ,$$

where *D* is the measured diffusion coefficient and *b* or *b* value is the diffusion encoding parameter (*γ* ^2^ *δ* ^2^ *g* ^2^(Δ − *δ*/3)), *γ* is the gyromagnetic ratio, *δ* is the length of the diffusion gradient pulses, Δ is the time interval between the gradients, and *g* is the amplitude of diffusion pulses [5]. The parameter kurtosis, *K*, is the normalized fourth moment of *P*(*r*) (the probability density function describing displacement *r*) [3, 4]:

2 $$K = \frac{{\int {r^{4} P(r){\text{d}}r} }}{{\left( {\int {r^{2} P(r){\text{d}}r} } \right)^{2} }} - 3.$$

Recently, the description of Eq. (1) for diffusion has found applications in imaging breast cancer [6], head and neck [7, 8], prostate [9], etc, because of better description of complex non-Gaussian diffusion and also better fitting results [10]. However, for any organ, fitting errors should be considered more accurately. In some cases, better fits does not mean that the model is better reflective of biophysical changes to a certain disease, because there might be substantial fitting errors associated with its parameters as will be shown in this study.

Kurtosis measurements in diffusion weighted imaging are made by acquiring images at multiple different *b* values and fitting the model to these signals with a variety of nonlinear least squares algorithms. Errors in measuring both *D* and *K* will depend both on noise in the signal and in the choice of *b* values. Previously, Fleysher et al. [11] determined the acquisitions that minimized noise in mono-exponential measurements with their results being applicable to selection of echo times in mono-exponential *T*_2_ relaxometry and selection of *b* values in mono-exponential apparent diffusion coefficient (ADC) measurements. In a similar study, Gilani et al. [12] minimized the noise in estimation of bi-exponential *T*_2_ measurements of the prostate cancer. In this study, the work was extended to optimize mono-exponential kurtosis measurements.

## Methods

All programs and numerical simulations were performed in MATLAB Release 2013b (The MathWorks, Inc., Natick, MA, US).

### Covariance Matrix in General

The signal is measured for multiple *b* values and the model is fitted using a variety of nonlinear, least squares fitting methods. The covariance matrix can be used to calculate the sensitivity of parameter estimates to each independent variable (i.e. *b* value) [12, 13]. Here, covariance matrix calculations were used to minimize errors in estimating parameters of mono-exponential kurtosis model.

In general, a function relating a set of measured signals, *y*_*i*_ (*i* = 1, 2, …, *m*), to a set of measurement parameters, *x*_*i*_ (e.g. the *b* values) is given by

3 $$y_{i} = f(x_{i} ;a_{1} ,a_{2} , \ldots ,a_{n} ),$$

where *a*_*j*_ (*j* = 1,2, …, *n*) are the parameters to be estimated (e.g. *D*, *K*, etc.).

The covariance matrix, **Q**, equals $({\mathbf{A}}^{\text{T}} \cdot {\mathbf{A}})^{ - 1}$ where **A** is an *m* × *n* matrix:

4 $$A_{ij} = \frac{1}{{\sigma_{0} }}\left( {\begin{array}{*{20}c} {\frac{\partial f}{{\partial a_{1} }}|_{{a,x_{1} }} } & \ldots & {\frac{\partial f}{{\partial a_{n} }}|_{{a,x_{1} }} } \\ \vdots & \ddots & \vdots \\ {\frac{\partial f}{{\partial a_{1} }}|_{{a,x_{m} }} } & \cdots & {\frac{\partial f}{{\partial a_{n} }}|_{{a,x_{m} }} } \\ \end{array} } \right).$$

*σ*_0_ is the acquisition noise which is assumed to be Gaussian, and equal for all acquisitions. This assumption is valid provided *σ*_0_ is less than about 0.25 (i.e. signal to noise ratio (SNR) is greater than 4), at which point the Rician nature of the noise becomes less apparent [14, 15]. For diffusion weighted imaging, SNR is usually defined as mean of signal divided by its standard deviation at *b* value of 0. Each column of **A** corresponds to one of the estimated parameters, and each row corresponds to a single measurement. Thus, *m* must therefore be greater than or equal to *n*. **Q**, is an *n* × *n* matrix and each diagonal element, **Q**_*ii*_, is the variance of the corresponding parameter *a*_*i*_ [13]. The coefficient of variation (CoV_*i*_), is therefore given by

5 $${\text{CoV}}_{i} = \frac{{\sqrt {{\mathbf{Q}}_{ii} } }}{{a_{i} }}.$$

The error in the *i*th parameter may be minimized by minimizing CoV_*i*_. Overall error is minimized by minimizing the mean square error (MSE), the trace of **Q**.

### Covariance Matrix of Kurtosis Model

For mono-exponential kurtosis, Eq. (1) could be rewritten with the style of Eq. (3) as follows:

6 $$S_{i} = f(b_{i} ;S_{0} ,D,K),$$

where *b*_*i*_ (*i* = 1,2, …, *m*) are the *b* values, *S*_*i*_(*i* = 1,2, …, *m*) are the measured signals at these *b* values, and *S*_0_, *D* and *K* are the three parameters to be estimated. Since there are three parameters to be estimated at least three (*b*_*i*_, *S*_*i*_) acquisitions are required.

The *m* × *3* matrix **A** for Eqs. (1) or (6) is:

7 $${\mathbf{A}} = \frac{1}{{\upsigma_{0} }}\left( {\begin{array}{*{20}c} {{\text{e}}^{{ - b_{1} D + \frac{{b_{1}^{2} D^{2} K}}{6}}} } & {S_{0} \left( { - b_{1} + 2\frac{{b_{1}^{2} DK}}{6}} \right){\text{e}}^{{ - b_{1} D + \frac{{b_{1}^{2} D^{2} K}}{6}}} } & {S_{0} \left( {\frac{{b_{1}^{2} D^{2} }}{6}} \right){\text{e}}^{{ - b_{1} D + \frac{{b_{1}^{2} D^{2} K}}{6}}} } \\ \vdots & \vdots & \vdots \\ {e^{{ - b_{m} D + \frac{{b_{m}^{2} D^{2} K}}{6}}} } & {S_{0} \left( { - b_{m} + 2\frac{{b_{m}^{2} DK}}{6}} \right){\text{e}}^{{ - b_{m} D + \frac{{b_{m}^{2} D^{2} K}}{6}}} } & {S_{0} \left( {\frac{{b_{m}^{2} D^{2} }}{6}} \right){\text{e}}^{{ - b_{m} D + \frac{{b_{m}^{2} D^{2} K}}{6}}} } \\ \end{array} } \right).$$

And finally, *Q* equals $({\mathbf{A}}^{\text{T}} \cdot {\mathbf{A}})^{ - 1}$. In principle it might be possible to derive **Q** analytically; however, this is not generally possible so that the calculations must be performed numerically over a discrete grid of *b* values. It was possible to organize the results based on encoding parameter *bD* meaning that the results are not dependent on *D* values.

*D* and *K* has been, respectively, measured to be around 0.86 ± 0.37 µm^2^ ms^−1^ and 1.5 ± 0.43 for head and neck tumours [8], or 2.51 ± 0.37 µm^2^ ms^−1^ and 0.57 ± 0.07, for healthy prostate and 1.55 ± 0.45 µm^2^ ms^−1^ and 0.96 ± 0.24 for cancerous prostate [9].

The optimization procedure could be performed to minimize errors in *D* alone, *K* alone or both of these parameters. This would require to minimize the *i*’th corresponding diagonal element of the covariance matrix, so that the coefficient of variation in estimating that parameter (${\text{CoV}}_{i} = \frac{{\sqrt {{\mathbf{Q}}_{ii} } }}{{a_{i} }}$) is minimized. If covariance matrix is derived from matrix of Eq. (7) then the second diagonal element of the covariance matrix corresponds to the variance in estimating parameter *D* and the third corresponds to the variance in estimating parameter *K*. Here to optimize both parameters *K* and *D*, the sum of CoV_2_ + CoV_3_ was minimized.

### Monte Carlo Verification

Monte Carlo simulations were used to confirm selected covariance matrix variations. Mono-exponential kurtosis model (Eq. (1)) was simulated, and either Rician or Gaussian noise (standard deviation 5 % of peak signal) was added. Obviously, for the case of Gaussian noise, at each *b* value a random value should be drawn from a Gaussian distribution with mean of zero and standard deviation derived from *S*_0_/SNR [*S*_0_ defined in Eq. (1)]. However, for the case of Rician noise signal at each *b* value is dependent on the acquired signal [14, 15]:

8 $$P(S_{n} (b)) = \frac{{S_{n} (b)}}{{\sigma^{2} }}{\text{e}}^{{ - (S_{{_{n} }}^{2} (b) + S^{2} (b))/2\sigma^{2} }} I_{0} \left( {\frac{{S(b) \cdot S_{n} (b)}}{{\sigma^{2} }}} \right),$$

where *S*(*b*) is the signal without noise (i.e. Eq. (1)) and *S*_*n*_(*b*) is the noisy signal at each *b* value. Accordingly, the Rician noise was constructed using MATLAB’s makedist program at each *b* value.

For both cases, after construction of noisy signal, a new mono-exponential kurtosis model was then fitted to the noisy signals using MATLAB’s nonlinear least squares curve fitting, lsqcurvefit. The procedure was repeated with 100,000 different sets of noise and the CoV of each parameter estimate calculated. These calculations were then repeated over the same n-dimensional grids of *b* values and optimized *b* values were found. 100,000 different values of *D*, *K* were stored and were used to calculate CoV_*D*_ and CoV_*K*_.

It is noteworthy that the covariance matrix optimization results for bi-exponential *T*_2_ imaging of the prostate, has already been verified by the Monte Carlo method in Gilani et al. [12].

### Maximum *b* Values

It is obvious that greater *b* values tend to minimize the error in estimating kurtosis parameter because the second term in the exponential ($Kb^{2} D^{2} /6$) which contains the kurtosis parameter is multiplied by *b* ^2^.

However, there are two maximum *b* value criteria. The first maximum *b* value limit is imposed by the scanner noise considerations. The second maximum limit is related to the fitting model. Jensen et al. [4]. calculated the maximum allowable *b* value for the mono-exponential kurtosis model to be 3/*DK*.

## Results

Table 1 summarizes the optimization results. The optimization is based on the encoding parameter *bD*. As observed in the table, at least one maximum *b* value is present in the optimized acquisitions.

**Table 1:** Optimum *b* value acquisition strategy to minimize estimation error of *D* and *K* for *N* (3–5) *b* value acquisitions, where $(bD)_{\hbox{max} } = 3/K$ is the maximum allowable acquisition point, ${\text{CoV}}_{K}$, ${\text{CoV}}_{D}$ and ${\text{CoV}}_{S}$ are the coefficients of variation, respectively, for *K*, *D* and *S*_0_

*N*	(*bD*)_1_	(*bD*)_2_	(*bD*)_3_	(*bD*)_4_	(*bD*)_5_	(*bD*)_max_	*K*	CoV_*K*_	CoV_*D*_	CoV_*s*_
3	0	0.75	2			2	1.5	0.145	0.220	0.05
3	0	0.9	3			3	1	0.153	0.188	0.05
3	0	1	3.75			3.75	0.8	0.176	0.177	0.05
3	0	1	5			5	0.6	0.245	0.168	0.05
3	0	1.05	6			6	0.5	0.335	0.164	0.05
3	0	0.9	10			10	0.3	1.48	0.173	0.05
4	0	0.8	0.8	2		2	1.5	0.145	0.171	0.05
4	0	0.95	0.95	3		3	1	0.153	0.144	0.05
4	0	1	3.75	3.75		3.75	0.8	0.126	0.176	0.05
4	0	1.05	5	5		5	0.6	0.173	0.166	0.05
4	0	1.05	6	6		6	0.5	0.237	0.163	0.05
4	0	0.95	10	10		10	0.3	1.04	0.164	0.05
5	0	0.8	0.8	2	2	2	1.5	0.108	0.168	0.05
5	0	1	1	3	3	3	1	0.118	0.142	0.05
5	0	1.05	1.05	3.75	3.75	3.75	0.8	0.122	0.133	0.05
5	0	1.1	1.1	5	5	5	0.6	0.173	0.125	0.05
5	0	1.05	6	6	6	6	0.5	0.194	0.1618	0.05
5	0	1	10	10	10	10	0.3	0.857	0.161	0.05

SNR = 20

To show the difference between the optimized results, the case of using equally distanced *b* values between 0 to maximum was also tested as shown in Table 2. Comparing Tables 1 and 2 it is clear that optimized *b* values considerably reduce the estimation errors of *D* and *K*.

**Table 2:** Equally distanced *b* value acquisition strategy for *N* (3–5) *b* value acquisitions, where $(bD)_{\hbox{max} } = 3/K$ is the maximum allowable acquisition point, ${\text{CoV}}_{K}$, ${\text{CoV}}_{D}$ and ${\text{CoV}}_{S}$ are the coefficients of variation, respectively, for *K*, *D* and *S*_0_

*N*	(*bD*)_1_	(*bD*)_2_	(*bD*)_3_	(*bD*)_4_	(*bD*)_5_	(*bD*)_max_	*K*	CoV_*K*_	CoV_*D*_	CoV_*s*_
3	0	1	2			2	1.5	0.145	0.223	0.05
3	0	1.5	3			3	1	0.15	0.22	0.05
3	0	1.88	3.75			3.75	0.8	0.18	0.24	0.05
3	0	2.5	5			5	0.6	0.24	0.29	0.05
3	0	3	6			6	0.5	0.34	0.36	0.05
3	0	5	10			10	0.3	1.48	1.13	0.05
4	0	0.66	1.33	2		2	1.5	0.14	0.19	0.05
4	0	1	2	3		3	1	0.14	0.17	0.05
4	0	1.25	2.5	3.75		3.75	0.8	0.16	0.17	0.05
4	0	1.66	3.33	5		5	0.6	0.22	0.19	0.05
4	0	2	4	6		6	0.5	0.30	0.22	0.05
4	0	3.33	6.66	10		10	0.3	1.29	0.50	0.05
5	0	0.5	1	1.5	2	2	1.5	0.13	0.18	0.05
5	0	0.75	1.5	2.25	3	3	1	0.14	0.16	0.05
5	0	0.94	1.86	2.81	3.75	3.75	0.8	0.15	0.15	0.05
5	0	1.25	2.5	3.75	5	5	0.6	0.21	0.16	0.05
5	0	1.5	3	4.5	6	6	0.5	0.28	0.17	0.05
5	0	2.5	5	7.5	10	10	0.3	1.11	0.31	0.05

SNR = 20

**Fig. 1:**
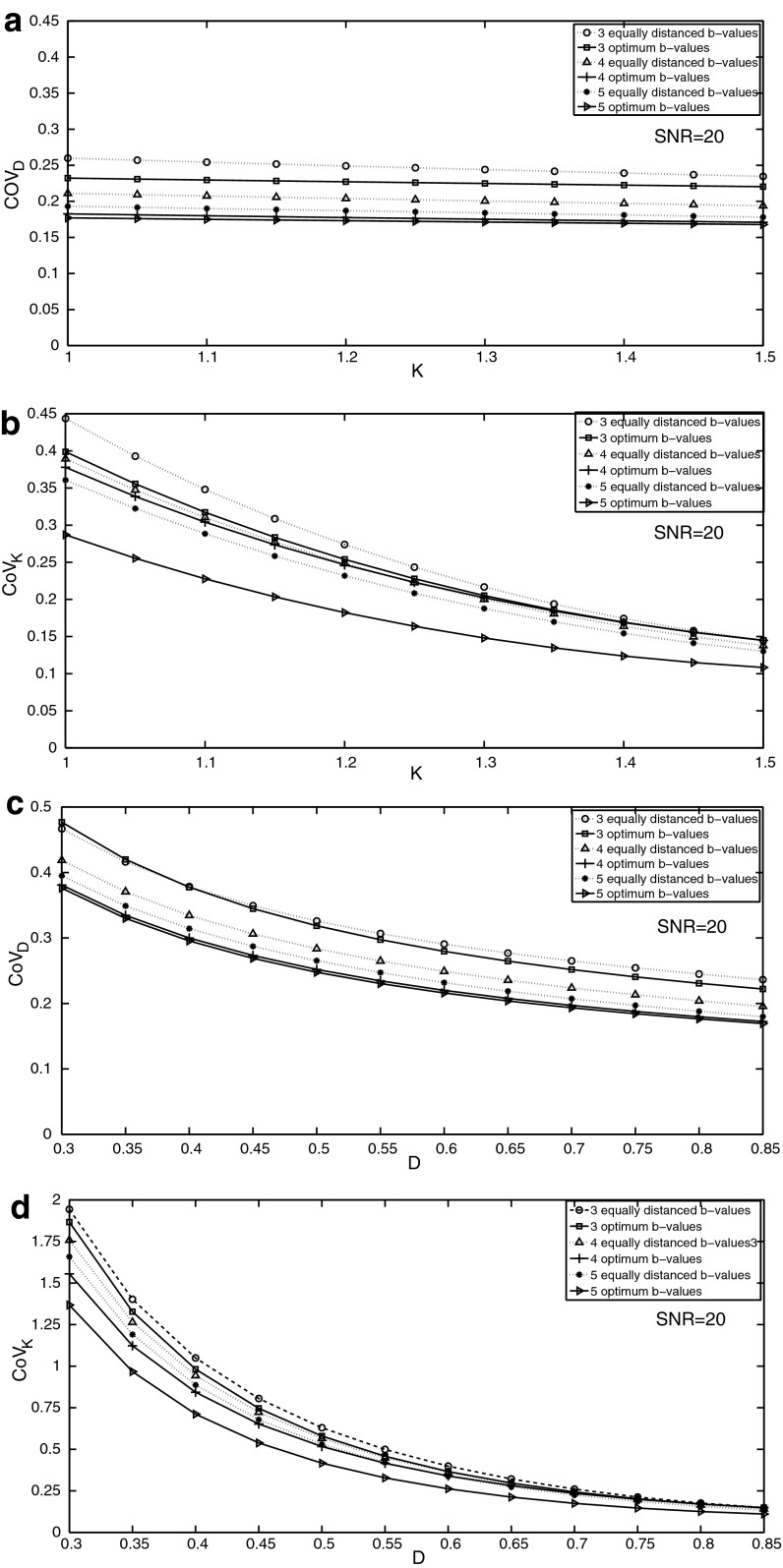
Changes in the coefficient of variation in estimating *D* and *K* (respectively, CoV_*D*_ and CoV_*K*_) with varying *K* and *D* for target values of head and neck tumours for 3–5 optimized or equally spaced acquisitions. **a** *K* varies from 1 to 1.5 and CoV_*D*_ is measured. **b** *K* varies from 1 to 1.5 and CoV_*K*_ is measured. **c** *D* varies from 0.3 to 0.85 and CoV_*D*_ is measured. **d** *D* varies from 0.3 to 0.85 and CoV_*K*_ is measured

To show a more tangible presentation of the optimization results, the values of *D* and *K* were selected to be 0.86 ± 0.37 µm^2^ ms^−1^ and 1.5 ± 0.43 similar to the values measured in head and neck tumour by Yuan et al. [8]. The optimization was performed using these target values, and estimation errors were compared with equally distanced acquisitions in Fig. 1 with varying *D* and *K*. In Fig. 1a, b, *D* was assumed to be constant and *K* varied from 1 to 1.5. In Fig. 1c, d, *K* was assumed to be constant and *D* varied from 0.35 to 0.85.

As observed in the four figures with increasing the number of *b* values above 5, it is always better to optimize the acquisitions than to use equally distanced *b* value acquisitions.

### Monte Carlo Verification

This procedure was repeated for all the target values of *D* and *K* in Table 1. CoV_*D*_ and CoV_*K*_ were found if Rician noises were present. Since here only SNR’s of 20 were considered, there was a good agreement between the Rician Monte Carlo simulations of noise and the results from covariance matrix calculations.

## Discussion

It was shown that as the number of *b* value acquisitions increases, optimization significantly reduces errors in measuring both diffusion and kurtosis. Although the optimizations are dependent on both parameters *D* and *K*, for one example which was the case of head and neck tumours, it was shown that the optimization works well for a wide range of these parameters. However, in addition to the maximum *b* value consideration regarding the kurtosis modelling, one should consider that if the both parameter *D* and *K* are small, then the maximum *b* value criteria is imposed by the maximum practical *b* values of the MR scanner. This means in many routine applications the maximum *b* values that are used are smaller than the maximums of Tables 1 or 2; hence there might be significant errors in estimating non-Gaussian parameters. For a clinical diffusion kurtosis imaging example where *D* is 1 μm^2^ ms^−1^ and *K* is 0.6, if five values of 0, 1000, 1500, 2000 and 2500 s mm^−2^ are used, CoV_*D*_ and CoV_*K*_ are, respectively, about 0.21 and 0.75 assuming SNR = 20.

There is an inverse relation between CoV’s derived in this study and SNR (${\text{CoV}} \propto \frac{1}{{\text{SNR}}}$). For example, if SNR is 30 instead of 20 then CoV’s derived here should be multiplied by 0.66. Parameter estimations are also dependent on diffusion echo time (*T*_E_) or signal fading due to *T*_2_ relaxation, similarly this can be accounted for by considering ${\text{CoV}} \propto \frac{1}{{{\text{e}}^{{ - T_{\text{E}} /T_{2} }} }}$.

Optimization and noise considerations of MR relaxometry acquisitions from a statistical point of view is not something new [16–21]. Gilani et al. [12] have optimized bi-exponential *T*_2_ measurements of prostate cancer. Jambor et al. [20] have optimized bi-exponential diffusion measurements of the prostate. Merisaari et al. [18] have optimized mono-exponential, bi-exponential and kurtosis measurements of the prostate, however, only for some random selections of *b* values instead of searching over a grid of *b* values. Additionally, optimization of diffusion kurtosis acquisitions has already been looked at in [22] calculating Cramér–Rao lower bound. In this study, the optimization was performed over a wide range of kurtosis values, applicable to diffusion kurtosis of many organs; also the whole allowable grid of *b* values was searched. Hence, our text gives a more direct and simplified assessment of nonlinear least squares curve fitting algorithm, which is routinely used for relaxometry.

In most of these studies, the Monte Carlo method is used. Monte Carlo method could directly assess the inherent fitting errors of any algorithm, and is relatively more accurate because the simulated noise could have exact profile of the acquisitions. For example, for diffusion weighted imaging with SNR’s of less than 4, the Rician nature of noise is more dominant and the Gaussian assumption is not valid [14, 15]; however, even in Monte Carlo optimizations of diffusion, still a Gaussian approximate of noise profile is used. This issue is not important for bigger region of interest (ROI) analysis because of significantly larger SNR’s, as a result, the Gaussian estimate of noise which was used in our study is valid.

Also, the covariance matrix method for estimating errors is fast. For example, if the number of *b* values is greater than 10, the optimization would be around 1 or 2 h using this method, whereas a similar Monte Carlo optimization would be significantly much more time consuming, and hence not feasible.

## Conclusion

The results of this study prove that using *b* values of as high as possible would significantly improve diffusion kurtosis imaging. However, there are two constraints for this, first it is the interference from higher terms of diffusion, and the second one is the noise limitations of the scanners at high *b* values.

Both covariance matrix calculations and Monte Carlo assessments of noise, predict the accuracy of diffusion parameter estimations and this could be used to optimize acquisitions. These might help in near optimal selection of *b* values, if target values of diffusion parameters are known.
